# The influence of structural disorder and phonon on metal-to-insulator transition of VO_**2**_

**DOI:** 10.1038/s41598-017-14235-w

**Published:** 2017-11-01

**Authors:** In-Hui Hwang, Zhenlan Jin, Chang-In Park, Sang-Wook Han

**Affiliations:** Department of Physics Education and Institute of Fusion Science, Jeonbuk(Chonbuk) National University, Jeonju, 54896 Korea

## Abstract

We used temperature-dependent x-ray absorption fine structure (XAFS) measurements to examine the local structural properties around vanadium atoms at the V K edge from VO_2_ films. A direct comparison of the simultaneously-measured resistance and XAFS regarding the VO_2_ films showed that the thermally-driven structural transition occurred prior to the resistance transition during a heating, while  this change simultaneously occured during a cooling. Extended-XAFS (EXAFS) analysis revealed significant increases of the Debye-Waller factors of the V-O and V-V pairs in the {111} direction of the R-phase VO_2_ that are due to the phonons of the V-V arrays along the same direction in a metallic phase. The existance of a substantial amount of structural disorder on the V-V pairs along the *c*-axis in both M_1_ and R phases indicates the structural instability of V-V arrays in the axis. The anomalous structural disorder that was observed on all atomic sites at the structural phase transition prevents the migration of the V 3d^1^ electrons, resulting in a Mott insulator in the M_2_-phase VO_2_.

## Introduction

Vanadium dioxide (VO_2_) is a typical metal-to-insulator transition (MIT) material, and it is accompanied by a first-order structural phase transition (SPT) from a monoclinic (M_1_) phase to a rutile (R) phase via a distorted-monoclinic(M_2_) phase. The MIT of VO_2_ is often compared with that of Ti_2_O_3_, which is known as a Mott-Hubbard MIT system with no structural transitions. VO_2_ have been extensively studied to understand the MIT mechanism^1–6^ and to develop its potential applications, including smart windows^7^, optical switches^8^, strain sensors^9^, and gas sensors^10^. Previous studies have showed that MIT in VO_2_ could be induced by various parameters, including thermal heating^1–6^, doping^11–13^, electric fields^14–16^, structural stress^9,17–19^, and photons^20–23^. The twist of V-O octahedra in the M_1_ and M_2_ phases and the dimerization of V-V pairs along the *c*-axis in the R phase, which are caused by strongly-correlated electrons, were proposed to understand the MIT in VO_2_^3,4,20–26^. Along with the co-workers, however, Qazilbash demonstrated a mixed  phase of insulating and metallic phases near the MIT temperature using infrared spectroscopy (IR) measurements. Recent neutron scattering studies showed a phonon contribution on the collapse of the bandgap^27^. Furthermore, metallic properties were observed even in the M_1_ phase near the MIT temperature^28^, even though the M_2_ phase was regarded as a Mott insulator. The decrease of the resistance in the M_1_ and M_2_ phases was ascribed to a percolation effect because a small portion of the metallic phase could be developed in the system. Tao *et al*. showed metallic properties that could be induced in the M_1_ phase via structural strain^29^, and other researchers have reported an observation of insulating properties in the R phase near the MIT temperature (T_c_)^17,30–33^. Thus, discussion on the origin of the MIT and the Mott insulator in VO_2_ is still ongoing.

A direct comparison of the electrical and local structural properties of VO_2_ provides important information in the attainment of an understanding of the MIT in VO_2_. Diffraction techniques are canonical methods that are used  to determine the structural properties of the crystals, and they can also be used to detect structural disorder in the Debye-Waller factor analysis. Transmission electron microscope (TEM) and scanning tunneling microscope (STM) measurements have been widely used to examine the atomic arrays in crystals. However, it  has not been easily to describe the local structural properties around a specific species atom in compounds. Transmission IR spectroscopy is a macroscopic tool that is limited in its detection of the local structural properties. The x-ray absorption fine structure (XAFS) analysis is a unique tool that can be used to describe the local structural properties around the atoms of a selected species; furthermore,  the XAFS can be easily adapted to other measurements. Previous studies of the XAFS on VO_2_, V_1−x_Cr_x_O_2_, and V_1−x_W_x_O_2_ reported local structural changes around the V and W atoms^3,11,33–35^. However, a direct comparison of the local structural and electric properties of the systems was not performed. For this study, simultaneous measurements of the XAFS at the V K edge and resistance from VO_2_ films were conducted to directly compare the structural and electrical properties. From in-situ XAFS measurements at the V K edge regarding VO_2_ films, the bond lengths and the Debye-Waller factors of the V-O and V-V pairs were quantitatively determined.  Extended XAFS (EXAFS) revealed an anomalous increase of the Debye-Waller factors of atomic pairs in the {111} direction of the R-phase VO_2_.

From the direct comparison of the *simultaneously* measured XAFS and resistance, the following findings were observed: 1) The SPT is congruent with neither the MIT nor the pre-edge peak shift during a heating, while the three transitions occur nearly at the same temperature during a cooling. 2) Insulating properties are evident in the R phase near the SPT. 3) The bond-length changes of the six V-O pairs on a V-O octahedron are non-rigid. 4) Two of the bonds of the V-O pairs are slightly longer than the other four bonds of a V-O octahedron in the R phase. 5) Anomalous structural disorder exists on all atomic sites at the SPT. 6) The Debye-Waller factor (σ^2^) of the V(0)-V(2) pairs along the {111} direction is larger by approximately 1.7 times in the R phase compared with that in the M_1_ phase, while on the V(0)-V(1) pairs along the c-axis, it remains at a constant value in the M_1_ and the R phases. 7) A substantial amount of structural disorder exists on the V(0)-V(1) pairs, compared to that on the V(0)-V(2) pairs.

## Results

**Figure 1:**
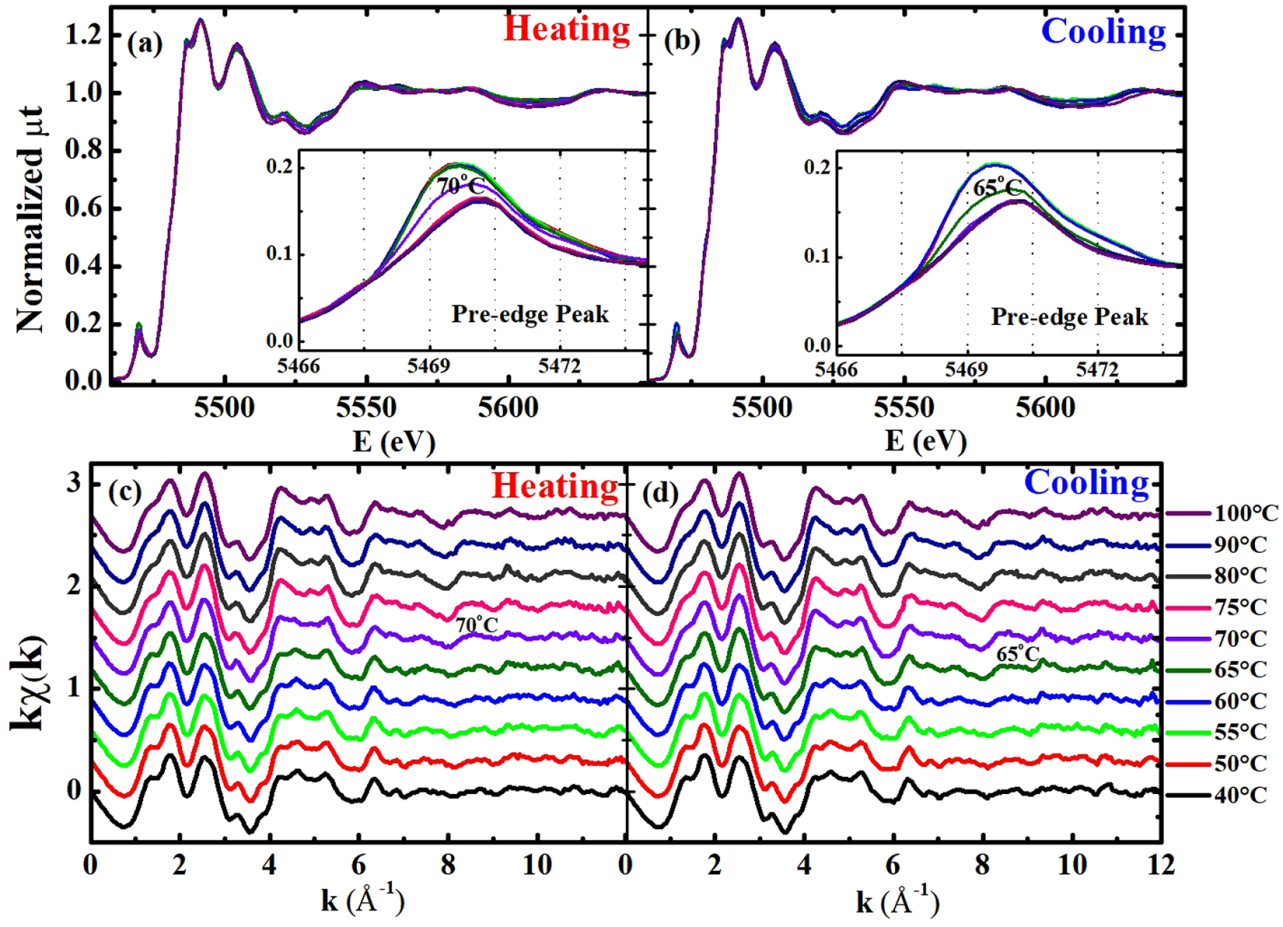
Normalized total x-ray absorption coefficient (μt) from the VO_2_ film at the V K edge as a function of the incident x-ray energy during (**a**) a heating and (**b**) a cooling from 40 to 100 °C. EXAFS (kχ) as functions of the photoelectron waver number, k, during (**c**) a heating and (**d**) a cooling. The EXAFS data in the range of 2.5–10.5 Å^−1^ were used for further analysis.

The x-ray absorption near edge structure (XANES) spectra at the V K edge demonstrated  a near-absence of change  in the main absorption edge, implying a constant chemical valence state of V^4+^ ions in the specimen, as shown in Fig. 1(a),(b). The pre-edge peaks near 5470 eV show the temperature-dependent behavior in both heating and cooling measurements, which is in sound agreement with previous studies^33–35^. The pre-edge peak mainly corresponds to a V 1 s → 3d quadrupole transition that hybridized with the V 4p orbitals, while the main edge near 5482 eV is determined by the V 4p states of VO_2_. The pre-edge peaks show transitions at 70 and 65 °C during heating and cooling processes, respectively. The pre-edge peaks at the transition metal K edges of the transition-metal oxides (TMOs) are mainly influenced by the 3d orbitals, the first neighboring oxygen atoms, and the second neighboring transition-metal atoms of the probing atom. The V 3d orbitals that split into the ${t}_{2g}$ and ${e}_{g}$ bands can be manifested in the pre-edge peaks at the V K edge. The details of the pre-edge peaks are discussed later and in the Supplementary Materials.

**Figure 2:**
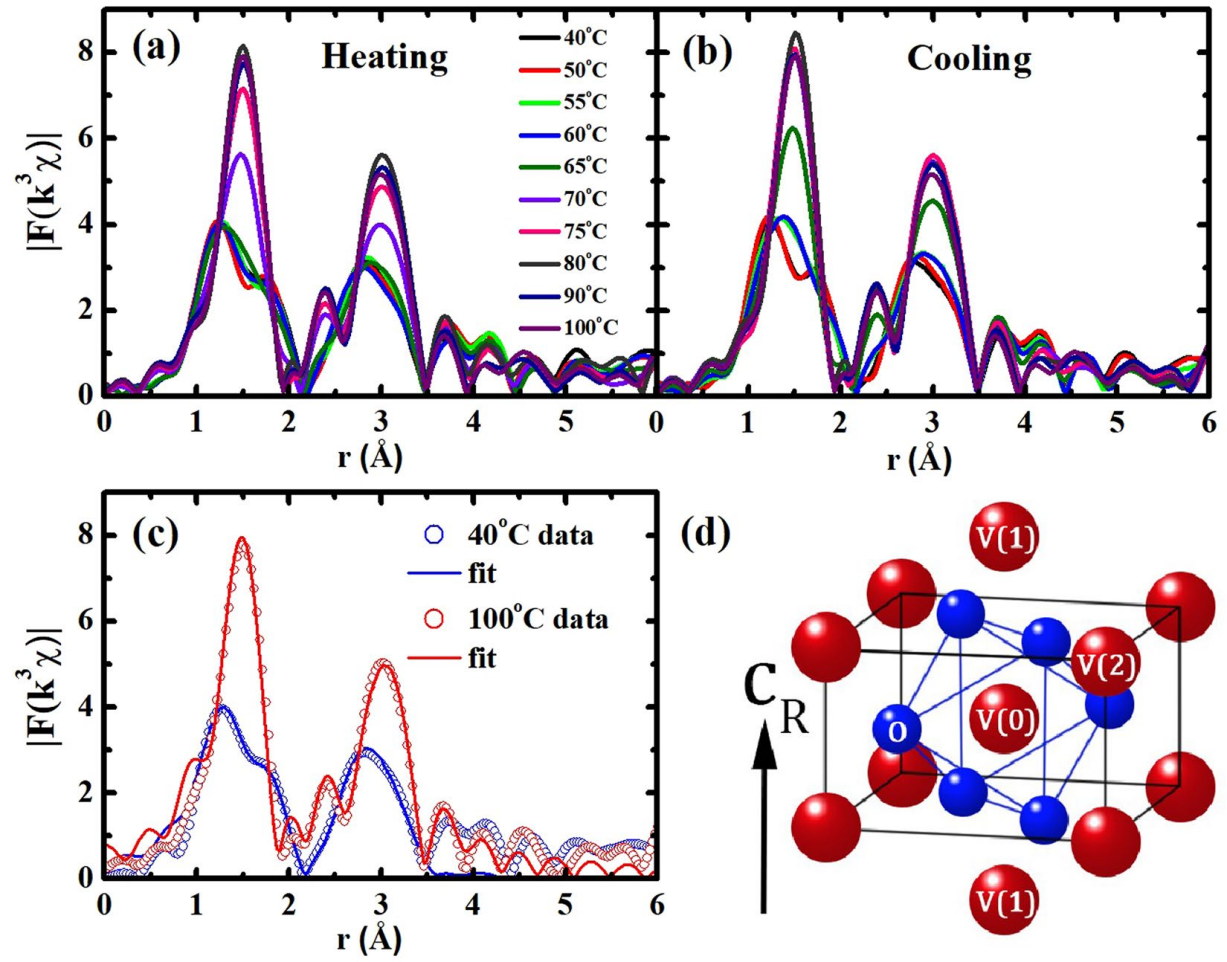
Magnitude of the Fourier transformed EXAFS as functions of the distance from a V atom during (**a**) a heating and (**b**) a cooling. For the Fourier transform, a Hanning window with a windowsill width of 1.0 Å^−1^ was used. (**c**) Representative fits of EXAFS data to the EXAFS theory. (**d**) The atomic positions around a core V(0) atom in the R-phase VO_2_.

In VO_2_, the first and second neighboring atoms of the V atom are six O and two V atoms, respectively. The O and V atoms are located omnidirectionally and along the *c*-axis in the R phase, respectively, as depicted in Fig. 2(d). The atomic distances and the Debye-Waller factors (σ^2^, including the thermal vibration and the static disorder) of the V(0)-O and V(0)-V pairs in VO_2_ can be quantitatively determined by analyzing the small oscillations (EXAFS) above the absorption edge, as can be seen in Fig. 1(a),(b) ^36,37^. The EXAFS data in Fig. 1(c),(d) were obtained, after the atomic absorption background was determined using AUTOBK (a part of IFEFFIT)^38^. The local structural changes can be more obviously elucidated in the Fourier transformed EXAFS in the *r*-space, as shown in Fig. 2(a),(b). The peak positions corresponding to atomic distances from a V atom in VO_2_ are approximately 0.3 Å shorter than the true atomic positions because the photoelectron phase shift has not yet been counted. The EXAFS data in the region of 1.0–3.5 Å were fitted in the *r*-space to the theoretical EXAFS calculations^39^ with the standard fitting procedures^40^. The fits include only the single scattering paths of the photoelectrons because the contribution of multiple-scattering paths to the EXAFS was negligible. The structural models of the M_1_ (space group P2_1_/c) and R (space group P4_2_/mnm) phases were used to fit the EXAFS data. The atomic positions of the R phase are shown in Fig. 2(d). The EXAFS data below and above 70 °C during the heating can be fitted only with the M_1_- and the R-phase models, respectively. Figure 2(c) shows representative EXAFS data and the best fits for the M_1_ and R phases. The details of the VO_2_ EXAFS data fit are discussed in the Supplementary Materials and elsewhere in the literature^33^.

**Figure 3:**
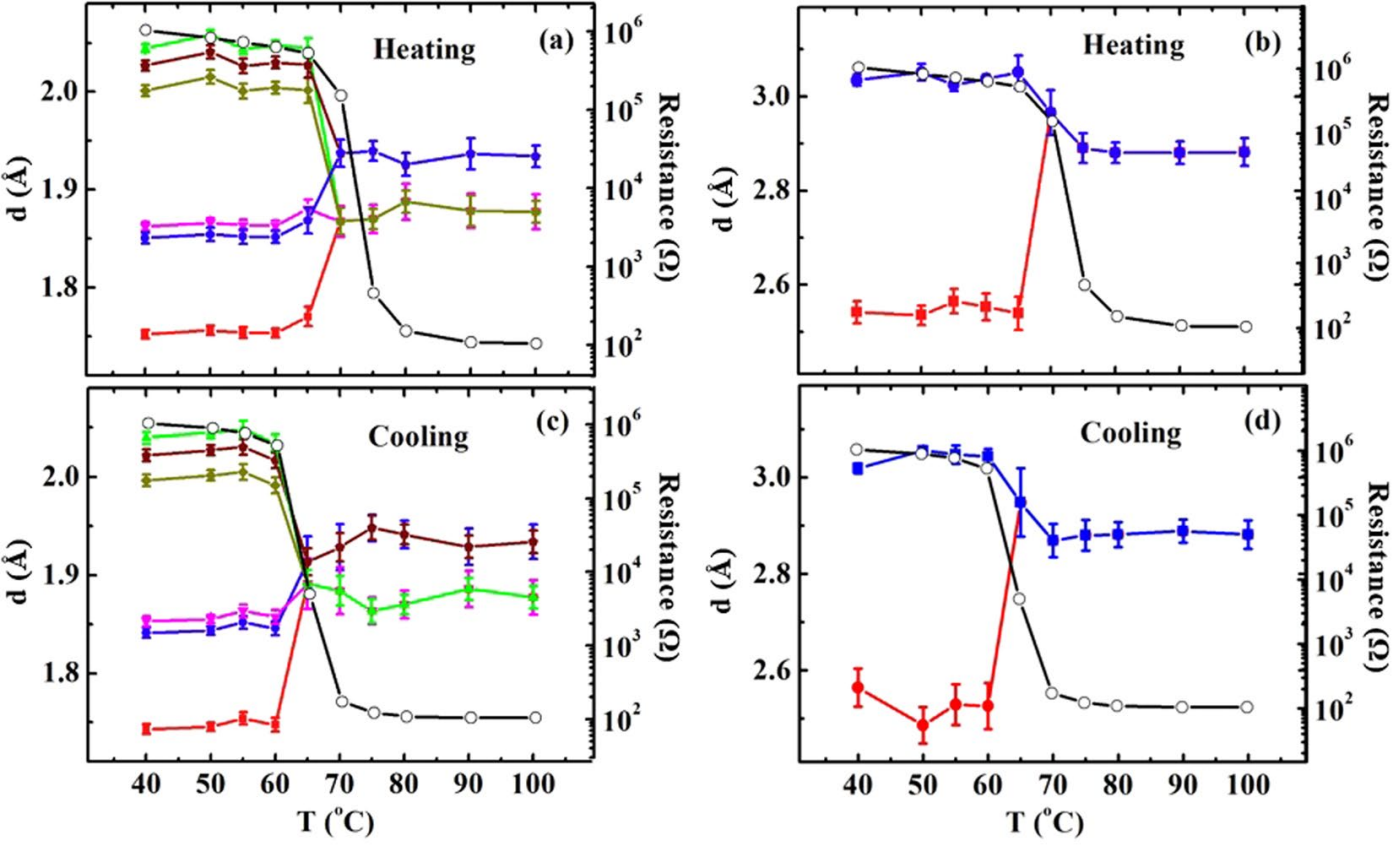
Atomic distances of (**a**), (**c**) V(0)-O and (**b**), (**d**) V(0)-V(1) pairs during the heating and cooling processes, respectively, as functions of the temperature with (black-open circles) *simultaneously*-measured resistance.

In Fig. 3, the temperature-dependent bond lengths of the V(0)-O and V(0)-V pairs that were obtained from the best fits are compared to the *simultaneously-*measured resistance. The EXAFS indicates the SPT that occurred between 65 and 70 °C during the heating. The SPT temperature is in sound agreement with the previous studies^3,11,28–30^. However, the SPT temperature does not correspond to the transition temperature of the pre-edge peak of 70 °C during the heating, as shown in Fig. 1(a). During the cooling, the pre-edge peak changes at 65 °C, while the local structure around the V atoms remains in the R phase and changes to the M_1_ phase at 60 °C. Previous studies demonstrated that a pre-edge peak at the transition-metal K edge is sensitive to the metal-metal pairs using *ab initio* calculations^41^. The EXAFS results in Fig. 3 indicate that the distances of the V(0)-V(1) pairs at 70 and 65 °C during heating and cooling, respectively, are in a middle value of the M_1_ (longer distance)- and R-phase values, even though the crystalline structure corresponds to the R phase. This result suggests that the pre-edge peak shift might be more sensitive to the distance of the nearest metals (V) rather than the SPT. A direct comparison of the pre-edge peak to the EXAFS obviously reveals that the SPT is prior to and lags behind the pre-edge peak shift during the heating and cooling, respectively.

**Figure 4:**
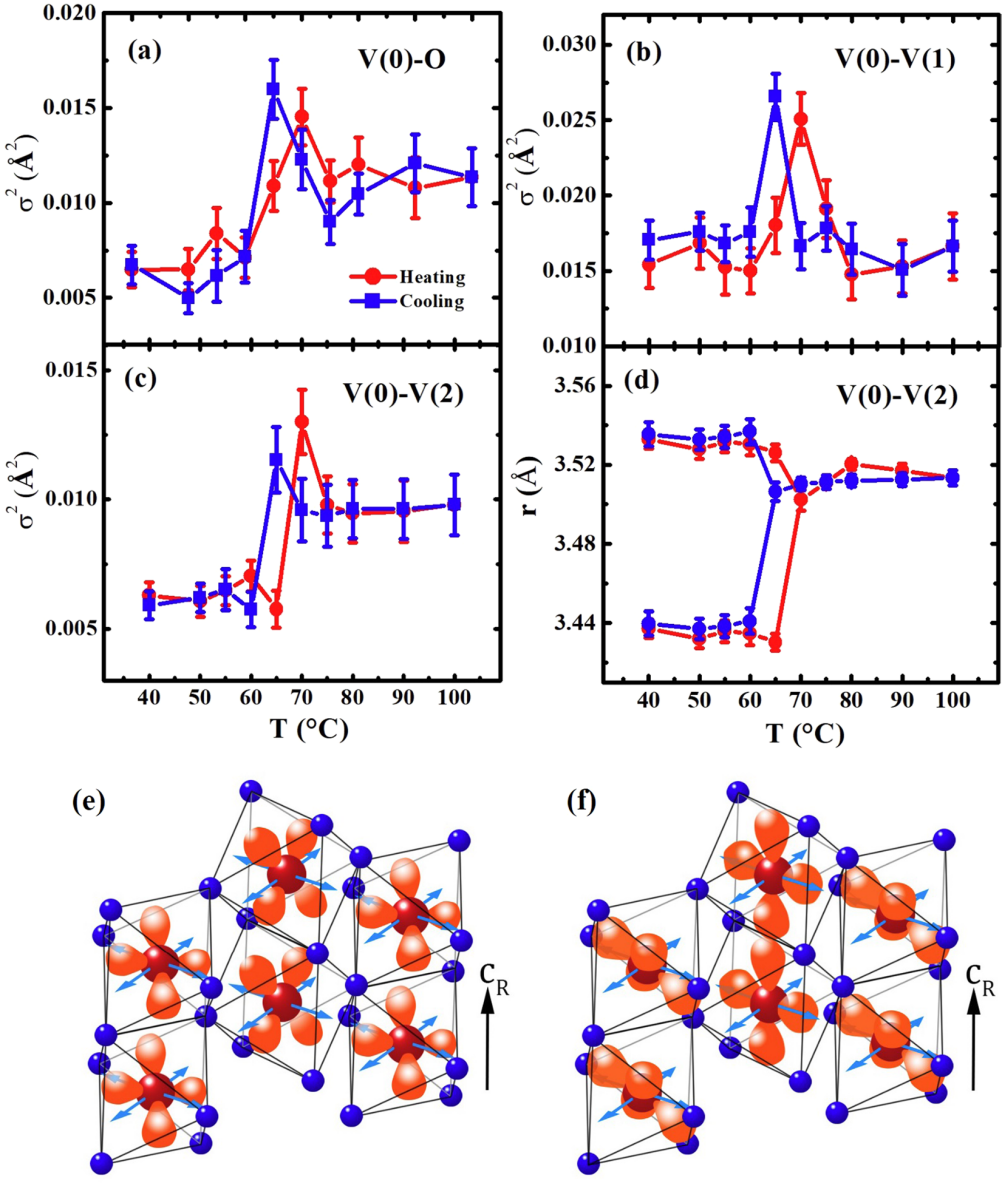
The temperature-dependent σ^2^s of the (**a**) V(0)-O, (**b**) the V(0)-V(1), and (**c**) the V(0)-V(2) pairs during (red dots) a heating and (blue squares) a cooling. (**d**) The temperature-dependent distance of the V(0)-V(2) pairs during a heating and a cooling. (**e**) and (**f**) The schematics of the ${{\boldsymbol{t}}}_{2{\boldsymbol{g}}}$(${{\boldsymbol{d}}}_{{\boldsymbol{xy}}}$, ${{\boldsymbol{d}}}_{{\boldsymbol{xz}}}$) and **d**_||_ of the V 3d orbitals of  V atoms in the (110) plane of the R-phase VO_2_, respectively, and the light-blue arrows indicate the {111} direction of phonon propagation.

The bond lengths of one, two, and three V(0)-O pairs are approximately 1.75, 1.85, and 2.01 Å, respectively, consisting of a distorted V-O octahedron in the M_1_ phase. The distance of the two V(0)-V(1) pairs are approximately 2.54 and 3.03 Å in the M_1_ phase, respectively. The distances of the V(0)-O and V(0)-V(1) pairs in the M_1_ phase are quite similar to those of previous reports^3,25,26,33^. The bond lengths of the six V(0)-O pairs cannot be fitted with a single-bond-length variable, strongly implying that the changes in the bond length are non-rigid. The non-rigid behavior of the V-O octahedra is in sharp contrast to the previous suggestions^3,25,26^. The M_2_ phase was proposed with a model of a half of the V(0)-V(1) pairs that are tilted and the rest are parallel-aligned along the *c*-axis of the R phase^17,25,26^; therefore, the bond lengths of the six V(0)-O pairs in the M_2_ phase are somewhat different from those in the M_1_ phase^25,26^. However, the EXAFS cannot distinguish the M_2_ phase from the M_1_ phase due to its resolution limit. In the R phase, the bond length of the two V(0)-O pairs is longer by ~0.06 Å compared with that of the four V(0)-O pairs in the V-O octahedron, as shown in Fig. 3(a),(c). The slightly-longer bond length of the apical O atoms in the TMO octahedra is a typical result of the crystal-field splitting of the 3 d orbitals into lower ${t}_{2g}\,$and higher ${e}_{g}$ bands that removes their degeneracy. The EXAFS result strongly suggests the splitting of the V 3 d orbitals into the ${t}_{2g}$ and ${e}_{g}$ bands, and the apical O atoms with a longer bond length from the central V atom of a V-O octahedron, that are crystallographically placed in a horizontal plane perpendicular to the *c*-axis of the R-phase VO_2_^25,26^. The V 3d^1^ electrons likely lie in a lower energy level of the ${t}_{2g}$(${d}_{xy}$, ${d}_{xz}$) band in the VO_2_, as illustrated in Fig. 4(e). The temperature-dependent atomic distances reveal the SPT temperatures differing from the T_c_s of the MIT during both heating and cooling. In the R phase near the SPT, the resistance shows that VO_2_ still remains in an insulating phase.

The mean Debye-Waller factors (σ^2^s) of the V(0)-O and V(0)-V(2) pairs as determined by the EXAFS-data fits are larger in the R phase than in the M_1_ phase, while, except at the SPT where the σ^2^ anomalously increases, they are nearly constant in the V(0)-V(1) pairs, as shown in Fig. 4. The previous studies theoretically and experimentally demonstrated that the R phase is structurally more stable than the M_1_ phase^42^. Thus, it is expected that the σ^2^ value of the V-V pairs will be larger in the M_1_ phase than in the R phase, because the zigzag pattern of the V atoms in the M_1_ phase can more effectively cause a static disorder in the atomic pairs, particularly the V(0)-V(2) pairs. Furthermore, an extra structural disorder is expected in the M_1_ phase because the R-phase VO_2_ films were initially synthesized at ~600 °C and cooled to the M_1_ phase. The thermal vibration and the static disorder, σ^2^(T) + σ^2^_static_, generally contribute to the σ^2^ of the atomic pairs, and σ^2^(T) can be understood by the Einstein or the correlated Debye model^40,43^. The abrupt increase of the σ^2^ of the V(0)-O and V(0)-V(2) pairs at the SPT is unexpected because the σ^2^(T) due to only the thermal vibration gradually increases during the heating. An extra σ^2^ value in the R phase may not come from a sudden increase in the structural disorder because the VO_2_ in the R phase is structurally more stable than that in the M_1_ phase^42^. The constant σ^2^ of the V(0)-V(1) pairs in the M_1_ and R phases is further evidence of the lack of any extra static disorder in the R phase. The extra σ^2^ of the V(0)-V(2) pairs in the R phase might correspond to the phonons that were observed with the use of neutron-scattering measurements^27^, and the extra σ^2^ of the V(0)-O pairs in the R phase can also be induced by phonons in V arrays along the {111} directions, because an O atom is located near the bonding line of the V(0)-V(2) pairs, and the EXAFS measures the motion of an O atom relative to a probing V atom. The absence of a change in the σ^2^ values of the V(0)-V(1) pairs in the M_1_ and the R phases indicates the lack of any extra phonons along the *c*-axis, and this result is in sound agreement with those of the previous studies^27^.

In the M_1_ phase, the σ^2^ of the V(0)-V(1) pairs is approximately 2.8 times larger than that of the V(0)-V(2) pairs. A large σ^2^ value in the V(0)-V(1) pairs indicates the existance of an extra structural disorder in the pairs over the entire temperature range because extra thermal phonons have not been observed along the *c*-axis^27^. The structural disorder in the V(0)-V(1) pairs likely prevents the propagation of the V 3d^1^ electrons along the *c*-axis, because previous studies reported that the electrical resistivity of metallic-metal oxides was increased due to a structural disorder^44^. The extra structural disorder at the SPT might be ascribed to the M_2_ phase in which the V(0)-V(1) pairs are partially parallel and tilted toward the *c*-axis^17,25,26^. However, the EXAFS cannot resolve a slight offset of an atomic position due to its resolution limit. The resistance from the VO_2_ films shows T_c_s values of ~73.8 and ~65.0 °C during the heating and cooling, respectively, as shown in Fig. 3. The heating T_c_ lags behind the SPT, while the cooling T_c_ is prior to the SPT. Based on a strongly-correlated-electron model^25,26^, it was expected that the resistance of the VO_2_ film would be considerably decreased in the R phase. However, in the R phase, the resistance decreases only slightly at 70 °C during the heating, and it increased at 65 °C during the cooling. The resistance in the R phase can be ascribed to a structural disorder. The anomalous structural disorders on all atomic sites at the SPT, as shown in Fig. 4, can effectively block the migration of the V 3d^1^ electrons, resulting in an inconsistency of the T_c_ and the SPT.

## Discussion

**Figure 5:**
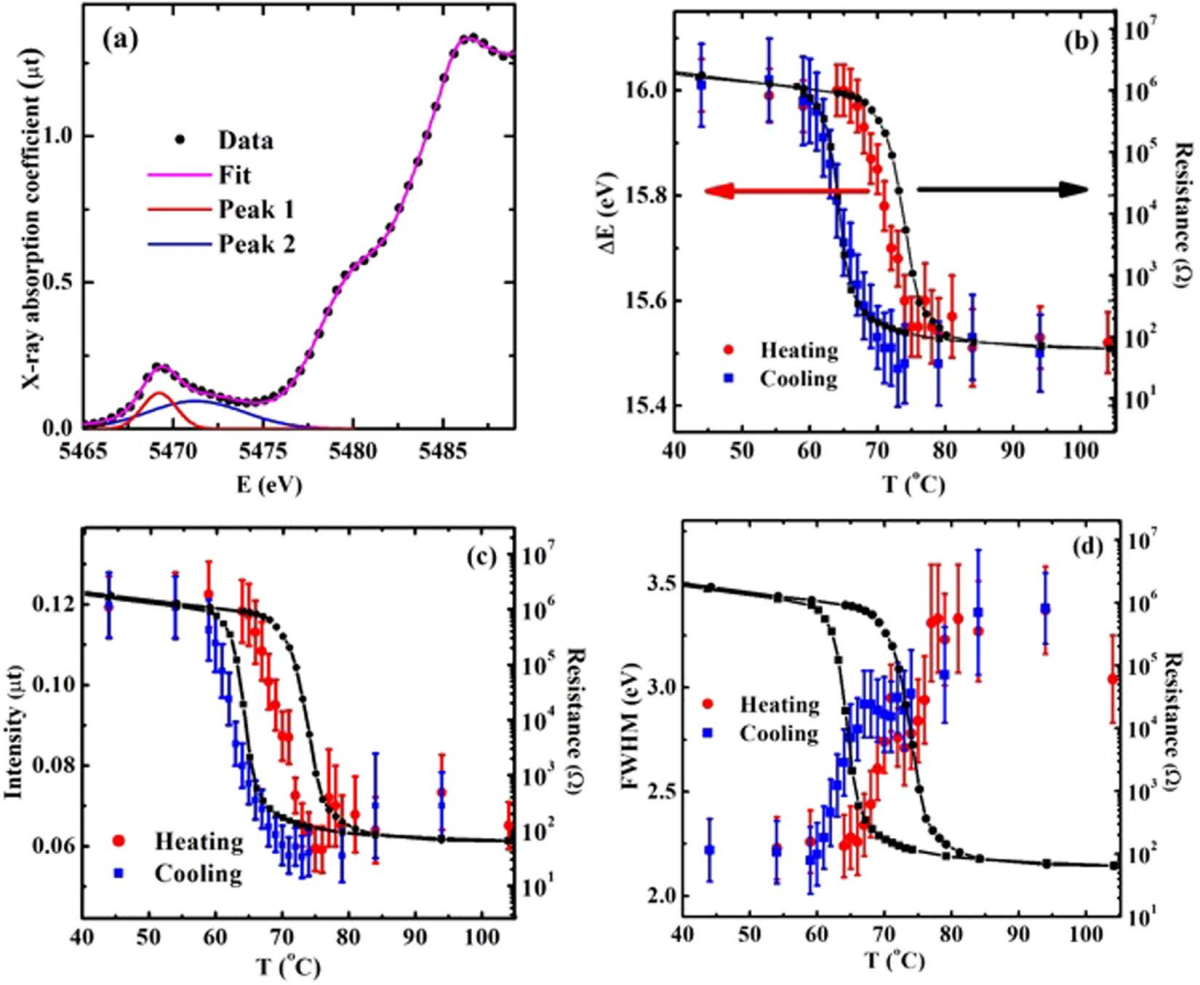
(**a**) XANES from the VO_2_ film at the V K edge and best fit with an arctangent-Gaussian model. (**b**) The relative position (ΔE) of the pre-edge peak (peak 1) to the main absorption edge, (**c**) the intensity, and (**d**) the FWHM of the first pre-edge peak with (black dots) *simultaneously-*measured resistance during (red dots) a heating and (blue squares) a cooling.

The metallic electrons are the V 3d^1^ electrons of VO_2_. The pre-edge peaks of the XANES at the V K edge reflect the local density of states (LDOS) of the V 3d orbitals. As mentioned above, the temperature-dependent behavior of the pre-edge peaks does not match with those of the SPT, as shown in Figs 1 and 3. The XANES was fitted to an arctangent-Gaussian model in which the pre-edge peaks are fitted with two Gaussian functions, as shown in Fig. 5 (a). Figure 5(b)–(d) demonstrate the relative position (∆E, E_main edge_ − E_pre-edge peak_), intensity, and FWHM (full-width at half maximum) of the first pre-edge peak(peak 1) at ~5469 eV that were obtained from the best fits, respectively. E_main edge_ of ~5482 eV is mainly determined by the V 4p states. The temperature-dependent behavior of the second pre-edge peak(peak 2) at ~5471 eV is discussed in the Supplementary Materials in detail. The ΔE change of ~0.5 eV before and after the transition temperatures is in sound agreement with the binding-energy change of the V 3d electron between the M_1_ and the R phases^45^. The separation of the pre-edge peaks corresponds to an energy gap between the ${t}_{2g}$ and ${e}_{g}$ bands^25,26^ that is reflected by the elongation of the apical oxygen distance in the V(0)-O octahedron, as determined by the EXAFS analysis. The measured separation of the pre-edge peaks of 2.0 ± 0.3 eV roughly agrees with the FEFF9 calculations (see Supplementary Materials) of ~2.8 eV and the band calculations of ~3.0 eV^26^. The pre-edge peaks imply that the Fermi level of VO_2_ lies within the first pre-edge peak as the result of only one electron in the V 3d orbitals.

The area of the pre-edge peaks is directly proportional to the local empty density of the states of the V 3d orbitals because the XANES detects the empty states near the Fermi level. The pre-edge peak becomes weaker and broader above the transition temperature, as shown in Fig. 5(c),(d). The intensity and the FWHM of the peak do not coincide with either the MIT or the SPT, and for the first pre-edge peak, it might be expected that it will become narrow when two bands in the M_1_ phase merge into a single band in the R phase. However, the peak broadens in the R phase compared to that in the M_1_ phase. These results strongly suggest that the changes of the LDOS of the V 3d orbitals are not directly proportional to the MIT. The area change of the first pre-edge peak that is obtained from the intensity and the FWHM of the peak cannot be precisely resolved due to a large fitting uncertainty. The transition temperatures of the resistance, the structure, and the pre-edge peak are summarized in Table 1. The pre-edge transition follows the SPT, and then the resistance changes during the heating, but these shifts occured nearly simultaneously during the cooling. The anomalous structural disorder that was observed near the MIT can play a critical role in the non-congruent transitions of the resistance, structure, and pre-edge peaks.

**Table 1:** The transition temperatures of the resistance, structure (EXAFS), and the ∆E, intensity, and FWHM of the pre-edge peaks from a VO_2_ film with an uncertainty of ± 0.5 °C determined by the best fits with an error function model.

	Resistance	Structure	∆E	Intensity	FWHM
Heating	73.8 °C	67.5 °C	71.5 °C	70.0 °C	68.5 °C
Cooling	65.0 °C	62.5 °C	65.0 °C	63.0 °C	63.5 °C

Theoretical calculations for which a dynamical mean-field theory (DMFT) was used demonstrated that the band gap in the M_1_ phase disappeared in the R phase^4^. However, other researchers have argued that the M_2_ phase that is evident near SPT is a Mott-Hubbard insulator^17,42,45^. Mott proposed impurity levels that collapse the bandgap in Ti_2_O_3_ above T_c_^46^. Hwang *et al*. demonstrated that an extra disorder in the Ti-Ti pairs in Ti_2_O_3_ plays an important role in the MIT^47^. The theoretical works did not include the structural disorder, the local distortion, and a non-rigid change in VO_2_ structure, particularly near the SPT^4,17,25,26^. This study elucidates that the insulating properties of the M_2_-phase VO_2_ are directly related to an anomalous structural disorder, particularly at the V sites. The significant influence of a structural disorder on the strongly-correlated electrons has been reported using various systems, including MIT materials^46,47^, superconductors^48–50^, Kondo effects^51^, and copolymers^52^. A direct comparison of the resistance and the XAFS measurements reveals that the insulating properties in the R-phase VO_2_ mainly originate from a structural disorder, while the metallic properties in the M_1_-phase VO_2_ could be ascribed to a percolation effect^2^ and a distortion of the V 3d orbitals^29^. A large amount of structural disorder on the V(1) sites in both the M_1_ and R phases indicates structural instabilities that can prevent phonon propagation and the V 3 d^1^ electron migration along the *c*-axis in the R phase.

The elongation of the apical O distance of the V(0)-O octahedron in the R-phase VO_2_ is evidence that the V 3 d^1^ electrons lie in the lower ${t}_{2g}$ band near the {111} direction (${d}_{xy}$ and ${d}_{xz}$ orbitals) of the R phase, as illustrated in Fig. 4(e). The V 3d^1^ electrons in the lower energy band of the ${d}_{xy}$ and ${d}_{xz}$ orbitals can jump to the higher energy band of the ${d}_{||}({d}_{{x}^{2}-{y}^{2}})$ orbital via a coupling with the phonons^22^, as illustrated in Fig. 4(f). The jump from the ${d}_{xy}$ and ${d}_{xz}$ orbitals to the ${d}_{\parallel }$ orbital was observed using photoinduced MIT measurements^22,23^. This can not only build a conduction channel along the *c*-axis, as depicted in Fig. 4(f), but, as illustrated in Fig. 4(e). the V 3d^1^ electrons can also jump to the next V atoms along the {111} direction with the mediation of the phonon in the same direction. For the latter case, the conduction electrons may migrate with a zigzag pattern along an external electric field direction; V(0) → V(2) → V(1) for an example of the external field in the *c*-axis. For the conduction electrons, a competitiveness between the (001)- and {111}-direction jumps can occure, because a considerable structural disorder is evident in the V(0)-V(1) pairs and the V(0)-V(2) distance is ~0.6 Å longer than the V(0)-V(1) distance in the R phase, as shown in Fig. 3 and 4. Thus, two-way (V(0)-V(1) dimerization along the *c*-axis and zigzag pattern) and one-way (zigzag pattern) channels can be main routes for the conduction electrons that migrate along the external electric field parallel and perpendicular to the *c*-axis, respectively. This scenario corresponds to the anisotropy conductivity of VO_2_, where higher and lower conductivity are parallel and perpendicular to the *c*-axis, respectively^14,24,53^. This study strongly suggests that the phonons in the {111} direction of the R-phase VO_2_ play a key role in the delocalization of the V 3d^1^ electrons, and that the structural disorder, particularly at V sites, prevents the propagation of electrons as well as phonons near the SPT temperature.

The position of the V atoms has a zigzag pattern in the M_1_- and M_2_-phase VO_2_. The zigzag pattern can not only suppress the degeneracy of the V 3d orbitals, but it can also spread the V 3d^1^ electrons onmidirectionally. In the M_1_-phaseVO_2_, the chance that the V 3d^1^ electrons can jump to the next V atoms is very slight due to the bandgap and the random direction of the orbitals. Pouget *et al*. reported that stress in the (110) direction of the R-phase VO_2_ affected the structural and electrical transitions more than that in the (001) direction^18^, suggesting the distorted-omnidirectional orbitals of the zigzag-patterned V atoms, thereby preventing the migration of the V 3d^1^ electrons^22,23^. When the crystals are released from the zigzag pattern, the V 3d orbitals are directionally aligned and the phonons can propagate along the {111} direction, reducing the total entropy^27^. On the condition that the V 3d orbitals are aligned in a certain direction, the vibration of the V atoms assists the delocalization of the V 3d^1^ electrons. In this scenario, the tetragonal symmetry in VO_2_ is a sufficient condition for its MIT. A static charge alignment along a certain crystalline direction in a tetragonal symmetry has been observed in various systems, including a static strip phase in La_2-x-y_Sr_x_Nd_y_CuO_4_^54^, polar tetragonal symmetry in BaTiO_3_^55^ and checkerboard phase in Ca_2-x_Na_x_CuO_2_Cl_2_^56^. The results of the present study strongly suggest that a parameter deriving a tetragonal symmetry in VO_2_ can induce its MIT without a bandgap change.

## Conclusion

Using the temperature-dependent XAFS measurements with simultaneously-measured resistance, it has been demonstrated that the SPT, the LDOS change of the V 3d orbitals, and the MIT do not occur at the same temperature during a heating, while the MIT nearly coincides with the SPT and LDOS change during a cooling. An anomalous structural disorder, particularly at V sites, effectively affects the migration of the metallic electrons, resulting in the Mott insulating properties in the M_2_ phase and the non-congruence of the SPT, MIT, and LDOS. The EXAFS measurements revealed a longer distance of the apical-O atoms of a V-O octahedron in the R-phase VO_2_, indicating the likely placement of the V 3d^1^ electron in the ${t}_{2g}$ band. With a tetragonal symmetry (R phase) to lower the entropy of VO_2_ at higher temperatures^27^, the phonons can propagate in V-atom arrays along the {111} direction. In the M_1_ phase, the V atoms are slightly offset from a diagonal line, so that phonon propagation is blocked. A large amount of structural disorder in the V(0)-V(1) pairs along the *c*-axis effectively prevents the phonon propagation. The thermally-induced phonons in the {111} direction assist the delocalization of the V 3d^1^ electrons in the R phase VO_2_ and the electrons likely migrate via the V-V array in the {111} direction as well as the V-V dimerization along the *c*-axis. This study clarifies that the tetragonal symmetry is essentially important for the metallic phase in VO_2_.

## Methods

### Synthesis of VO_2_ films

The *b*-oriented VO_2_ films were synthesized on α-Al_2_O_3_(0001) substrates via DC-magnetron-sputtering deposition. A vanadium target (purity: 99.9%) was used. The base and working presses of the sputtering chamber were ~10^–6^ and ~10^−3^ Torr under an Ar atmosphere, respectively. The substrate temperature was maintained at ~500 °C with a sputtering power of ~30 W. The films were annealed at ~500 °C for ~30 minutes under a mixture gas flow of Ar and O_2_ with an Ar: O_2_ flow ratio of ~5: 1. The synthesis of VO_2_ films was described elsewhere in detail^33^.

### Characterization

The *b*-oriented VO_2_ films with a lattice constant *b* of 4.785 Å were evaluated using x-ray diffraction measurements with conventional tube x-rays with Cu K_α_ radiation in air at room temperature. The mean grain size of ~2 μm and thickness of ~0.10 μm of the films were observed via field-emission scanning electron microscopy (SEM, S-5500, Hitachi).

### XAFS measurements and analysis

XAFS measurements were performed from VO_2_ films at the V K edge (5465 eV) by selecting the incident x-ray energy with a three-quarters tuned Si(111) double crystal monochromator at the 8 C beamline of the Pohang Light Source (PLS) during the heating and cooling in the temperature range of 40–100 °C. In the fluorescence mode at the incident x-ray angle of 45° to the film surface, the XAFS measurements of the VO_2_ films were *simultaneously* carried out with resistance measurements by using a home-made *in-situ* cell to directly compare the results of these two sets of measurements. At least three XAFS scans were taken at each temperature to exclude any unexpected error during the measurements. The temperature of the specimen during the XAFS scans was precisely monitored and controlled within an error of ± 0.1 °C, and the self-absorption of the films with a thickness of ~0.10 μm is negligible because the one-absorption length of VO_2_ at the V K edge is ~6.5 μm. The XAFS data were analyzed using the IFEFFIT package^38^ and standard EXAFS fitting procedures^40^. The theoretical calculations of EXAFS and LDOS were obtained using the FEFF9 code^39^.

### DC resistance measurements

The DC resistance of the VO_2_ films was measured by using a two-probe system at an applied voltage of 0.5 V in the home-made *in-situ* cell. The XAFS and the resistance measurements were performed after the system temperature stabilized because the resistivity of the films showed a time-dependent behavior just after being heated or cooled^33^.

## Electronic supplementary material

Supplementary information 
