# Supplementary material for: The influence of structural disorder and phonon on metal-to-insulator transition of VO2
Source: Sci Rep. 2017 Nov 1;7:14802. doi: 10.1038/s41598-017-14235-w (PMC5666023; doi:10.1038/s41598-017-14235-w)
Supplement: Supplementary file 1 — Supplementary information [file 41598_2017_14235_MOESM1_ESM.pdf]

Supplementary Materials

The influence of structural disorder and phonon on metal-to-insulator transition of VO_2

In-Hui Hwang, Zhenlan Jin, Chang-In Park, and Sang-Wook Han

1. XANES Analysis

The XANES spectra correspond to electron transitions from deep levels to empty states near the Fermi level of the selected element in a system. XANES at a transition metal K edge reflects the local density of states (LDOS) of the metal 3d orbitals and the local structural properties of neighboring atoms around the transition metal atom. The main absorption edge near 5482 eV that is shown in Fig. 1 corresponds to the 1s→4p transition of the electrons of the vanadium (V) atoms in VO_2 and it is considerably sensitive to the geometry of six oxygen (O) atoms around a V atom. The pre-edge peaks near 5469 eV in Fig. 1 are proportional to the empty density of the V 3d states because the V 3d states hybrid with the V 4p states, although the V 1s→3d transition is considerably weak due to the quantum selection rules. The previous studies have showed that the pre-edge peaks of a transition metal are also substantially affected by the second nearest neighboring atoms of the metal atoms[41]. In VO_2, a V-O octahedron consists of one central V atom and six O atoms as the nearest neighbors of the V atom. In the M_1 and M_2 phases, the V-O octahedron has a distorted form with different bond lengths and tilted angles of the six V-O pairs. Meanwhile, it becomes a correct octahedron in the R phase. The local structural changes around a V atom considerably affect the main absorption edge and also contribute to the pre-edge peaks because the distances and the geometries of the V-O and the V-V pairs change due to the SPT.

Figure S1(a),(b) present the normalized XANES spectra, $\mu t(E)$, at the V K edge during a heating and a cooling, respectively. The pre-edge peaks are sharp and dull below and above a transition, respectively. The temperature-dependent pre-edge peaks have a hysteresis behavior during the heating and cooling process. The main absorption edge also shows a temperature-dependent behavior. The quantitative changes of the pre-edge peaks and the main absorption edge were determined by fitting data to an arctangent-Gaussian model. The pre-edge peaks were fitted to two Gaussian functions by varying the positions, intensities, and FWHM (full-width at half maximum) of the pre-edge peaks, as shown in Fig. 5(a)-(d). The positions for both first and second pre-edge peaks are nearly constant within the range of uncertainty in the temperature range of 30 - 110°C, although they slightly move toward higher energy in the R phase, as shown in Fig. S2(a),(b). This finding indicates few changes in the energy levels of the V 3d states during the heating and the cooling processes. The relative position (ΔE) that is shown in

Fig. 5(b) was obtained from the positions of the main absorption edge and the first pre-edge peak. The main absorption edge is mainly contributed by the V 4p levels and the geometry of the nearest neighboring O atoms in VO_2. The ΔE mainly corresponds to the change of the main edge position because the position of the first pre-edge peak did not change much in the temperature range of 30 - 110°C, as shown in Fig. S2(a). The temperature-dependent behavior of the main absorption edge position could be ascribed to a change in the local structure, including the bond lengths, the Debye-Waller factors, and the geometry of neighboring atoms around the V atoms.

The temperature-dependent behaviors of the second pre-edge peak are shown in Fig. S2(b)-(d). The position and the FWHM of the peak remained at nearly constant values in the temperature range of 30 - 110°C, as shown in Fig. S2(b),(d). The intensity of the second pre-edge peak in the R phase was decreased by approximately 40%, compared to that in the M_1 phase. The second pre-edge peak corresponds to the empty states of the V 3d e_g band. The intensity decrease of the second pre-edge peak in the R phase implies that V $3d^1$ electrons filled the e_g band because the FWHM of the peak was nearly constant, resulting in the decrease of the peak area. The electrons in the e_g band could play an important role in the metallic phase VO_2 because the e_g band lies just above the Fermi energy level. The transition temperatures of the peak intensity are approximately 75°C and 69°C during heating and cooling, respectively, as shown in Fig. S2(c). These values correspond to none of the transition temperatures which are shown in Table 1, although they are relatively closer to the transition temperatures of the resistivity. The analysis results of the second pre-edge peak show that the state change of the e_g band does not correspond to either the SPT or the change of the main absorption edge.

XANES does not directly reflect the LDOS due to the quantum selection rules. The LDOS at the V K edge for the VO_2 were calculated using FEFF9 code[39] with the crystalline structures of the M_1 and the R phases that were determined by the best fits of EXAFS. Figure S3(a) shows the calculated x-ray absorption coefficient spectrum near the V K edge, XANES, for 27 atoms in a cluster with the diameter of ~1.2 nm. The calculated XANES spectra for the M_1 and the R phases are comparable to the measured XANES. The calculations show that the first pre-edge positions of both M_1 and R phases appear at ~5469 eV and that the Fermi levels are placed at -12.016 eV and -12.254 eV for the M_1 and the R phases, respectively. Lowering the Fermi level corresponds to raising the first pre-edge peak position, therefore, the energy gap between the positions of the main absorption edge and the first pre-edge peak decreases in the R phase, compared to that in the M_1 phase. This calculation is in sound agreement with the measurements that are shown in Fig. 5(b). The LDOS of the V 3d orbitals is nearly independent of the M_1 and the R phases, as shown in Fig. S3(b). The two peaks at -10.2 and -8 eV in Fig. S3(b) correspond to the pre-edge peaks near 5472 eV in Fig. S3(a). The constant value of the peak positions of the V 3d

orbitals in the M_1 and the R phases agrees well with the measurements that are shown in Fig. S2(a),(b). Figure S3 (c),(d) present the LDOSs of the s and p orbitals in the M_1 and R phases, respectively, that considerably affect the main absorption edge and the whiteline. The LDOSs of the s orbitals in both phases are quite similar to each other but are different in the p orbitals, particularly near $\Delta E = 7$ eV that corresponds to the whiteline in the absorption spectra. The calculations are evident that the SPT does not significantly contribute on the changes of the LDOSs.

2. EXAFS Analysis

The main contributors to EXAFS(χ) are neighboring atoms around a probing atom[36,37]. After the atomic background was determined using AUTOBK[38], the EXAFS was extracted from the raw data as a function of the photoelectron wave number, $k = \sqrt{2m(E - E_0)}/\hbar$, where m is the electron mass, E is the incident x-ray energy, E_0 is the absorption edge energy, and ħ is the Planck constant, as shown in Fig. 1 (c),(d) and Fig. S4 (a),(b). The EXAFS data in the range of $k = 2.5 – 10.5$ Å^{-1} were used for the Fourier transform to the r-space. The EXAFS data in the region of 1.0 – 3.5 Å were fitted in the r-space. The Fourier-transformed EXAFS consists of two parts, real and imaginary, as shown in Fig. S4 (c),(d), and these correspond to the EXAFS magnitudes in Fig. S4 (c),(d), respectively. The real and imaginary EXAFS parts were *simultaneously* fitted to the EXAFS theoretical calculations[39] using the same variables. The EXAFS(χ) is theoretically described, as follows[36,37,S1]:

$$\chi(k) = -\sum_j 3(\hat{\varepsilon}\cdot\hat{R}_j)^2 \frac{S_0^2\ N_j F_j(k,R_j)}{kR_j^{\,2}} e^{-2k^2\sigma^2_j} e^{-2R_j/\lambda(k)} \sin[2kR_j + \Phi_j(k,R_j)], \qquad (1)$$

Where $\hat{\varepsilon}$ is the electric field direction of the incident x-rays, S_0^2 accounts for the change of passive photoelectron waver functions in the presence of a core hole and multi-excitations, N_j is the mean coordination number, F_j is the photoelectron back-scattering amplitude, R_j is the jth shell atomic distance, σ_j^2 is the relative distance disorder (Debye-Waller factor) between the probing atom and the jth shell atoms, Φ_j is the total global phase shift of the photoelectrons from the probing atom and the jth shell atoms, and λ is the effective mean free path of the photoelectrons.

The F_j, λ, and Φ_j values of each atomic shell in Eq. (1) were theoretically calculated using the FEFF9 code[39] with the given atomic positions that were calculated using the ATOMS code[38]. The EXAFS data were fitted to the EXAFS theoretical calculations of F_j, λ, and Φ_j using the IFEFFIT software[38]. In the EXAFS fits, the atomic distance (R) and the Debye-Waller factor (σ^2) of each atomic shell and the absorption-edge energy (E_0) initially varied. Here, the presence of vacancies on the V and O sites in the VO_2 films was not assumed. Since the interest of the authors comprises the structural properties of the V-O and V-V pairs, the EAXFS data in the region of 1.0 – 3.5 Å were fitted, as indicated by the vertical-

dashed lines in Fig. S5. Due to the high correction of the V(0)-O pairs in the fitting region of 1.0 – 2.2 Å, the three and two bond lengths varied for the M_1 and the R phases, respectively, fixing the other bond lengths with the offset values. Each of the σ^2 variables for the six V(0)-O pairs and the two V(0)-V(1) pairs was used. The E_0 was initially fitted and finally fixed at the best value that was determined by the best fit because of its correlation to the other variables; therefore, ten and seven variables were in the final fits for the M_1 and R phases, respectively, as shown in Table S1. All of the independent data points of the fits were estimated as approximately 12.7 using Stern's rule[S3], as $N = \frac{2\Delta R\Delta k}{\pi} + 2$, where $\Delta R = 2.5$ Å (fitted region) and $\Delta k = 8.0$ Å^{-1} (Fourier-transformed region). The freedoms of the fits were 2.7 and 5.7 for the M_1 and the R phases, respectively. The quality of the fits was determined using the *R*-factor and a reduced-χ^2, where the goodness-fit criteria of the *R*-factor is < 0.02 and the reduced-χ^2 is ~1[S2,S3]. After satisfactory fits were obtained, the atomic-distance and σ^2 values of the V(0)-O and V(0)-V pairs were determined.

Figure S5 (a),(b) show the real EXAFS part and the best fits of the six V(0)-O, two V(0)-V(1), and eight V(0)-V(2) pairs at 40 and 100°C, respectively. It is evident that the EXAFS in the regions of 1.0 – 2.2, 1.7 – 2.7, and 2.5 – 3.5 Å mainly correspond to the V(0)-O, V(0)-V(1), and V(0)-V(2) pairs, respectively, although a moderate overlap region is also evident. It is worth noting that the peak positions are approximately 0.3 Å shorter than the true atomic distances because the total global phase shift of Φ_j in Eq. (1) is not considered here. The true distance of each atomic pair can be obtained from a goodness fit because the total global phase shift is theoretically counted in the fit. The magnitude of the Fourier-transformed EXAFS presents more convincing atomic positions, as shown in Fig. S5 (c),(d). However, the EXAFS magnitude degraded the structural information compared to the real and imaginary parts. The distance and the σ^2 of each atomic pair were independently determined using the best fits of the EXAFS data that were measured at a certain temperature. Table S1 summarizes the representative fitting results of the EXAFS data at 40 and 100°C. The temperature-dependent distances and σ^2 values that are shown in Fig. 3 and 4 were determined from the best fits of the EXAFS data at different temperatures using the same manner that is mentioned previously.

References

[S1] Han, S.-W. X-ray absorption fine structure and nanostructures. Int. J. Nanotech. **3**, 396-413 (2006).

[S2] Jeong, E.-S. *et al*. Local Structural Properties of CuI at Low Temperatures. J. Phys.: Condens. Matter. **23**, 175402 (2011).

[S3] Han, S.-W. *Fundamentals of XAFS*. (Baleon Pub. Co., 2016).

Table S1. The results of EXAFS data fits of the VO_2 film at 40 and 100°C, as shown in Fig. 3 and 4. S_0^2 of 0.8 that was determined using a *k*-weight fit[S2] of EXAFS data from a VO_2 powder[33] was fixed for the other fits of EXAFS data from VO_2 films. In the fits of the M_1 phase, there were four variables (3 *d*s and 1 σ^2) for the V(0)-O pairs, three variables (2 *d*s and 1 σ^2) for the V(0)-V(1) pairs, and three variables (2 *d*s and 1 σ^2) for the V(0)-V(2) pairs, while in the fits of the R phase, there were three variables for the V(0)-O pairs, two variables for the V(0)-V(1) pairs, and two variables for the V(0)-V(2) pairs.

T(°C)	V(0)-O(1)		V(0)-O(2)	V(0)-O(4)	V(0)-V(1)		V(0)-V(1′)	V(0)-V(2)		V(0)-V(2′)
	d (Å)	σ^2 (Å^2)	*d* (Å)	*d* (Å)	*d* (Å)	σ^2 (Å^2)	*d* (Å)	*d* (Å)	σ^2 (Å^2)	*d* (Å)
40	1.752	0.006	1.851	2.001	2.541	0.015	3.034	3.437	0.006	3.534
100	1.877	0.011	1.877	1.934	2.881	0.017	2.881	3.513	0.010	3.513

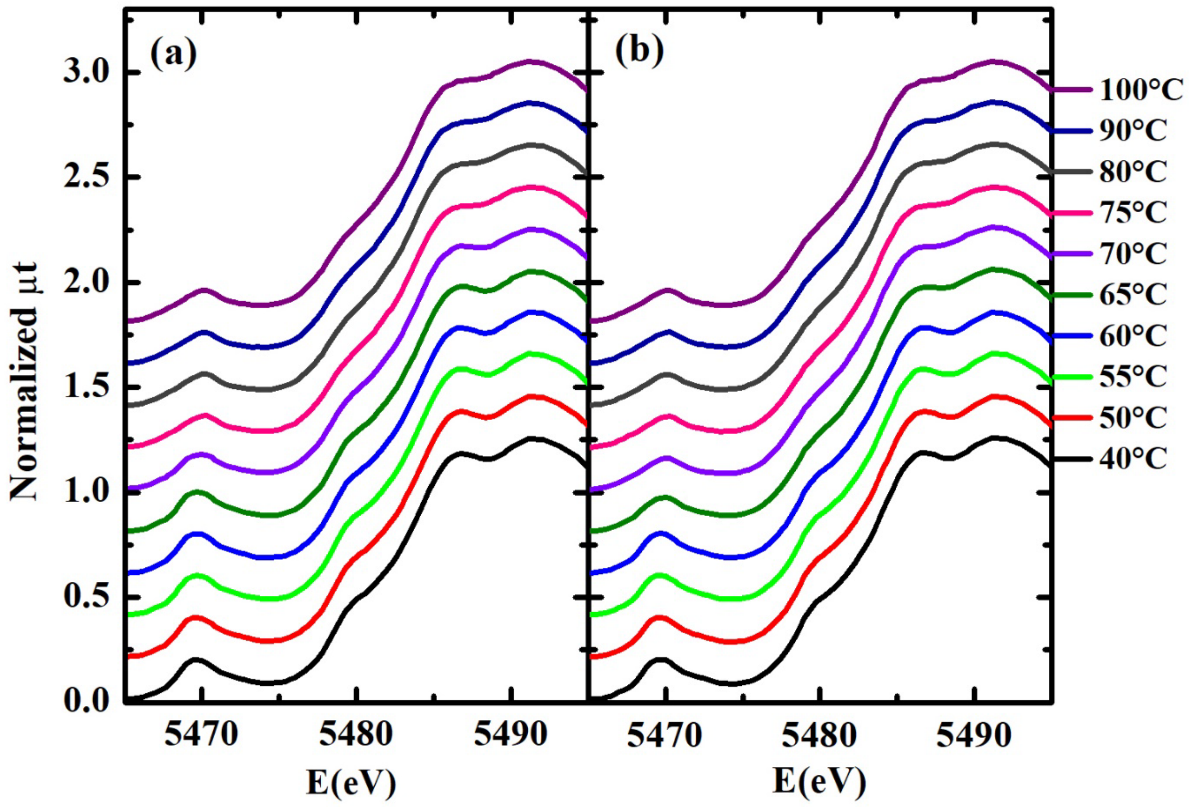

Figure S1. Normalized total x-ray absorption coefficient (μt) from the VO_2 films at the V K edge during (a) a heating and (b) a cooling as functions of the incident x-ray energy at different temperatures.

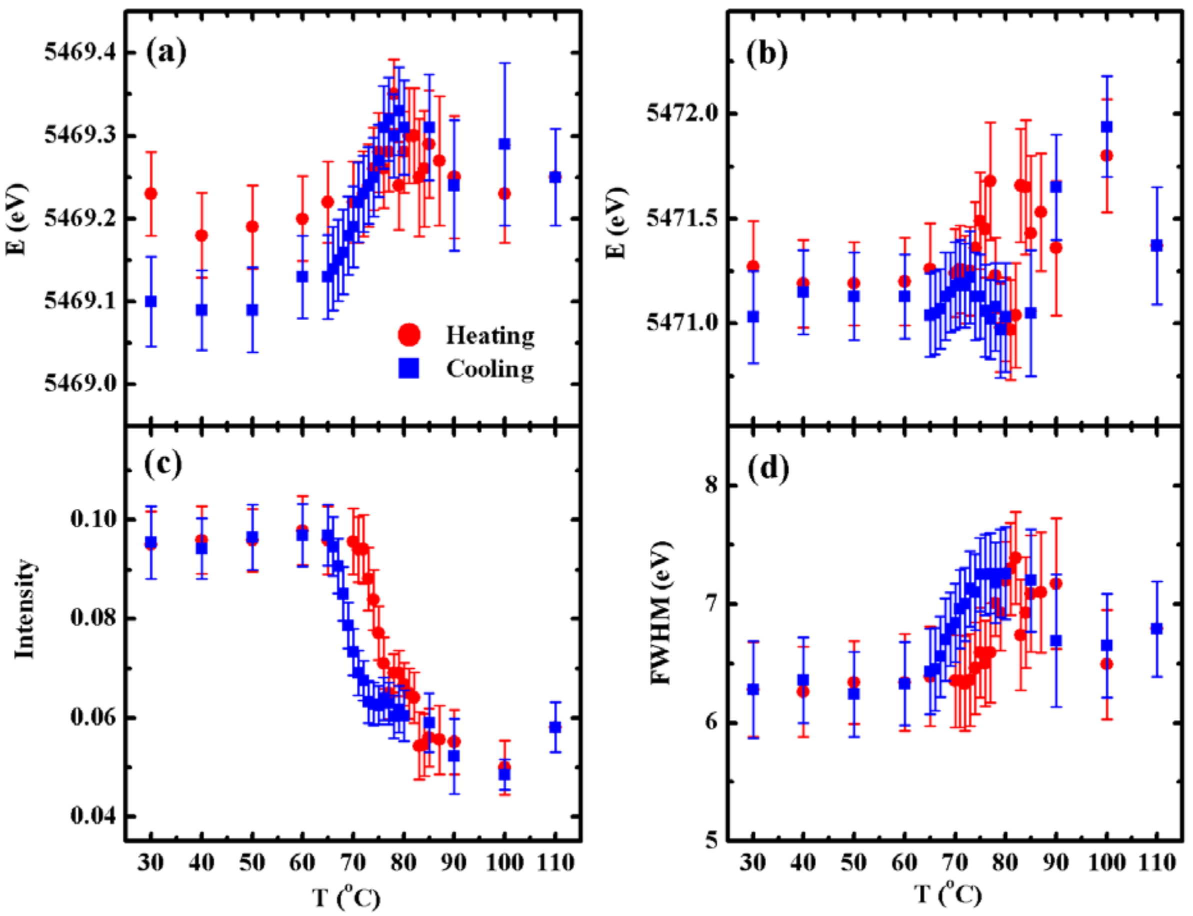

Figure S2. The fit results of the XANES. (a) and (b) The positions of the first and the second pre-edge peaks, respectively, as functions of the temperature during (red dots) a heating and (blue squares) a cooling. (c) and (d) The temperature-dependent intensity and FWHM of the second pre-edge peak, respectively.

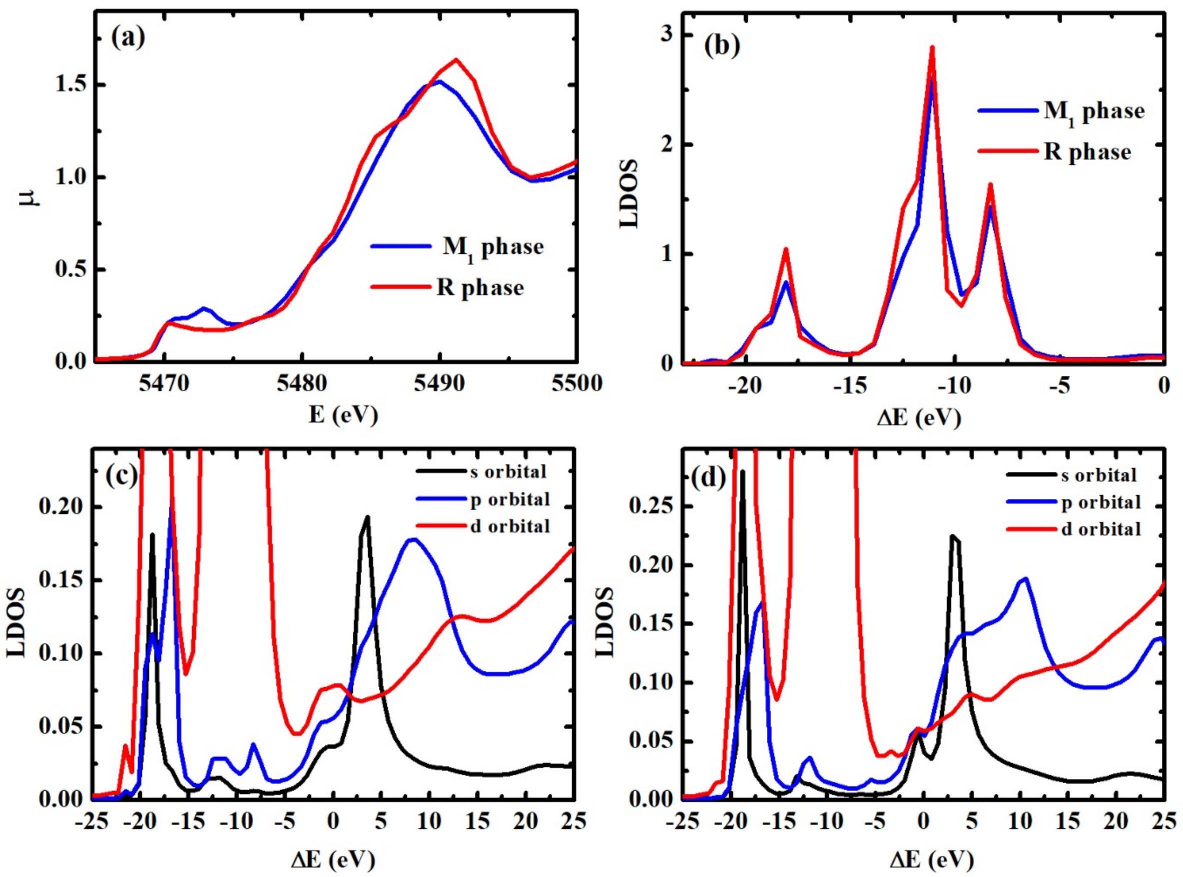

Figure S3. (a) Calculated x-ray absorption coefficient (μ) spectra, XANES spectra, for the M_1 and the R phase VO_2 at the V K edge using the FEFF9 code. (b) The LDOS of the V 3d orbitals near the V K edge. (c) and (d) The LDOS of the V s, p, and d orbitals at the V K edge for the M_1 and the R phase VO_2, respectively.

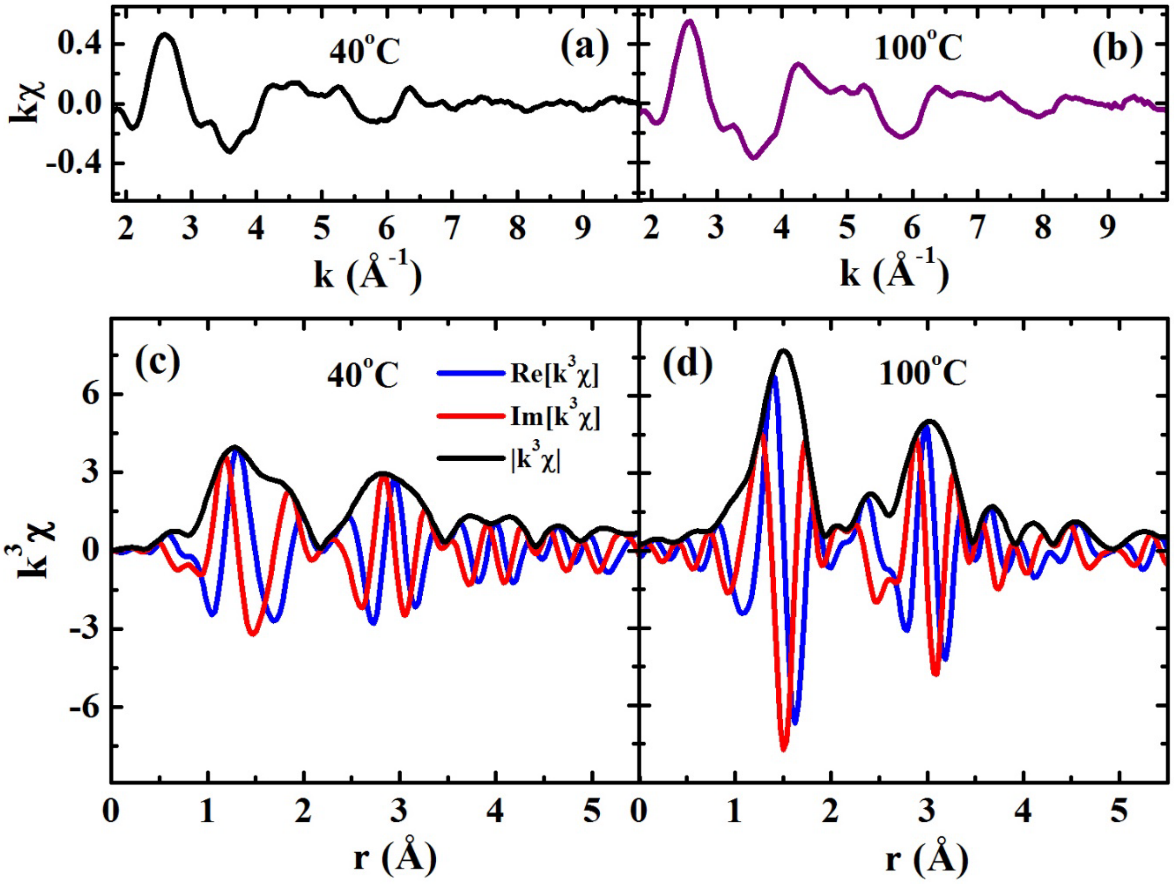

Figure S4. (a) and (b) EXAFS(kχ) at 40 and 100°C, respectively, as functions of the photoelectron wave number, k, described in the text. (c) and (d) The Fourier-transformed EXAFS($k^3\chi$) of (blue line) real, (red line) imaginary, and (black line) magnitude at 40 and 100°C, respectively, as functions of the distance from a V atom.

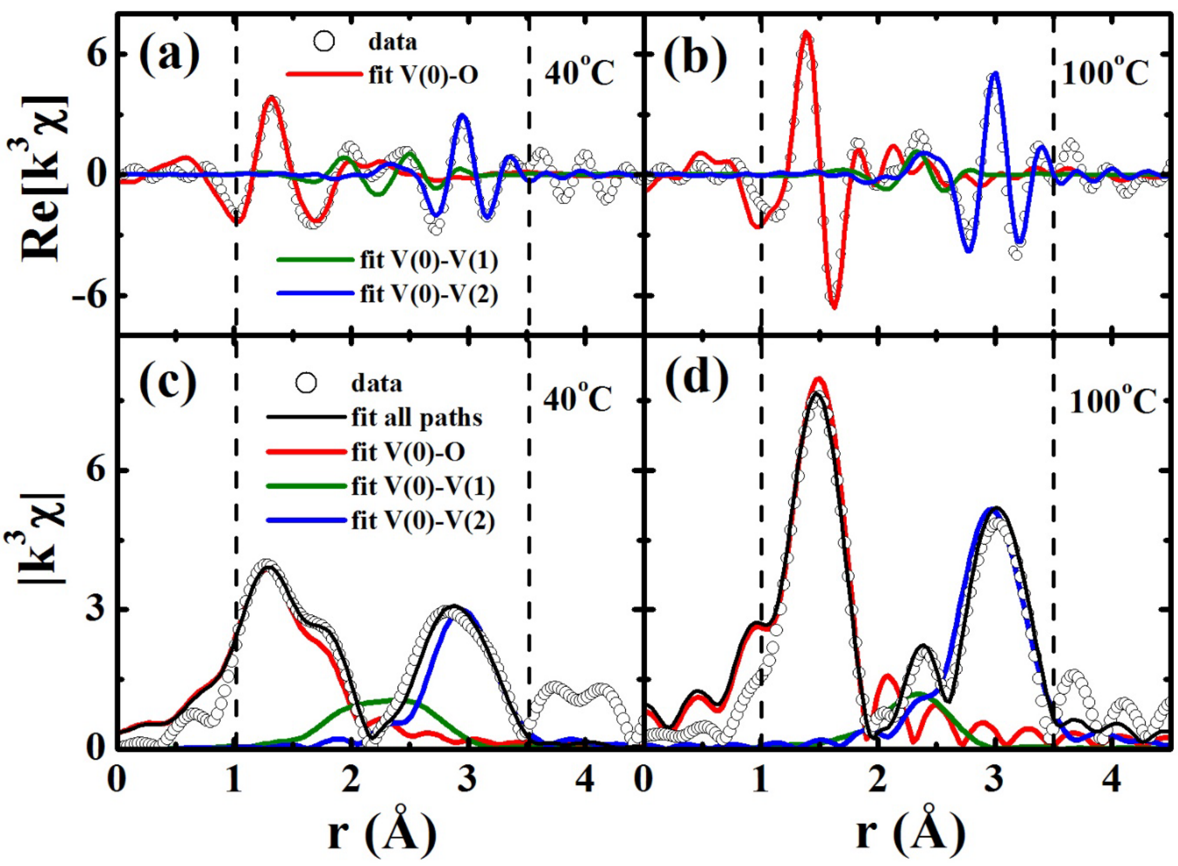

Figure S5. (a) and (b) The real parts of the EXAFS ($k^3\chi$) and (c) and (d) the magnitude of the EXAFS ($|k^3\chi|$), (open circles) data and the best fits of (black line) all, (red line) six O, (green line) two V(1), and (blue line) eight V(2) atoms, as functions of the distance from a V atom at 40 and 100°C, respectively. The vertical-dashed lines indicate the regions of the fits.
